# Night-to-Day Ratio Specified by 24-hour Blood Pressure Monitoring, Arterial Stiffness and Cardio-Ankle Vascular Index as Predictive Factors of Cardiovascular Risk

**DOI:** 10.33549/physiolres.935593

**Published:** 2025-10-01

**Authors:** Alena HAVELKOVA, Petr DVORAK, Michal POHANKA, Petr DOBSAK, Jarmila SIEGELOVA, Germaine CORNELISSEN

**Affiliations:** 1 Department of Sports Medicine and Rehabilitation, St. Anne’s Faculty Hospital, Brno, Czech Republic; 2 Department of Physiotherapy, Faculty of Medicine, Masaryk University, Brno, Czech Republic; 3 Department of Biology and Wildlife Diseases, Faculty of Veterinary Hygiene and Ecology, University of Veterinary Sciences, Brno, Czech Republic; 4 Halberg Chronobiology Center, University of Minnesota, Minneapolis, MN, USA

**Keywords:** Ambulatory blood pressure monitoring, Night-to-Day blood pressure ratio, Ambulatory Arterial Stiffness Index, Cardio-Ankle Vascular Index, Ankle Brachial Index, Ischemic heart disease

## Abstract

This study compares the interrelationships among different methods of determining predictive factors of cardiovascular risk: the Night-to-Day ratio (ND-R), Ambulatory Arterial Stiffness Index (AASI), Cardio-Ankle Vascular Index (CAVI), and Ankle-Brachial Index (ABI). A total of 8120 blood pressure measurements were obtained from 280 24h-ABPM records (29 values per daily record) of 20 patients who each provided two 7-day/24-hour monitoring sessions. For each of the two 7-day-24h-ABPM records, the ND-R and AASI were determined. CAVI and ABI were always examined at the beginning of each 7-day-24h-ABPM session. All 20 patients (12 men; 8 women; mean age 57±2.1 yrs; mean BMI 29.3±1.69 kg/m^2^; mean left ventricle ejection fraction 53±3.8 %) had chronic ischemic coronary artery disease. The correlation coefficients did not exceed 0.318. ND-R of SBP showed the highest methodological sensitivity, identifying 65 % of patients at increased risk, compared to 57.5 % for ND-R of DBP, 23.7 % for CAVI, and 2.5 % for AASI (up to 27.5 % by evaluating individual days). The different cardiovascular risk assessment methods (ND-R, AASI, CAVI and ABI) cannot be substituted for one another. No risk was demonstrated using ABI. Repeating the 7-day-24h-ABPM approximately 1 year apart (unless there is a change in medication or in clinical symptoms) revealed a significantly different results of the ND-R and AASI, which can be expected in approximately 25 % of patients.

## Introduction

Cardiovascular morbidity is the main cause of all death in developed countries. Another factor contributing to further increase of cardiovascular mortality is ageing. There are various approaches how to assess cardiovascular risk. Measurement of blood pressure (BP) belongs to set of basic clinical examinations. However, routinely used ambulatory BP measurement is not appropriate due to several reasons, white-coat-syndrome being one of them. To overcome such drawbacks, BP can be monitored over 24 h (24h-blood pressure monitoring, 24h-ABPM). Less often employed, however very valuable approach, represents 7-day ambulatory blood pressure monitoring (7-day-24h-ABPM) [1]. Analyses of obtained BP curves revealed significant information not only about BP values, but also about BP variability [2].

Among others, two parameters can be calculated based on the obtained BP curves. First, Night to Day Ratio (N-DR, %) is obtained by dividing the difference between the mean daytime and mean nighttime BP measurements by mean daytime BP. Second, the Ambulatory Arterial Stiffness Index (AASI) is calculated as 1 – the regression slope of diastolic blood pressure (DBP) on systolic blood pressure (SBP). This parameter reflects the relation between DBP and SBP depending on the structural and functional characteristics of large arteries [3].

Another possibility to study vascular conditions is the measurement by vascular screening device VaSera. Two parameters are extracted from such measurement: the ankle-brachial index (ABI), which is the ratio of SBP measured at the ankle to SBP measured at the brachial artery. Originally described by Winsor in 1950, this index was initially proposed for the non-invasive diagnosis of lower-extremity peripheral artery disease [4,5]. In 1980, Hayashi *et al.* [6] proposed to calculate the stiffness parameter β=1n(Ps/Pd)·D/ΔD, where Ps is SBP, Pd is DBP, D is the diameter of the artery, and ΔD is the change in arterial diameter between SBP and DBP. The cardio-ankle vascular index (CAVI) was established with the objective of obtaining an arterial stiffness index that is not affected by BP at the time of measurement, and which reflects the stiffness of a considerable length of the artery [7]. CAVI reflects the stiffness of the whole arterial segment composed of the aorta, femoral artery, and tibial artery, and can be calculated from the pulse wave velocity (PWV) at the origin of the aorta to the ankle portion of the tibial artery, and SBP and DBP measured at the upper brachial artery. This index was originally derived from the stiffness parameter β [8]. A more recent classification divides risks into five quintiles (quintile 1, ≤7.55; quintile 2, 7.60–8.20; quintile 3, 8.25–8.80; quintile 4, 8.85–9.45; and quintile 5, ≥9.50). The cumulative incidence of adverse cardiovascular events is significantly higher for CAVI>9.5 (5^th^ quintile) than for CAVI<7.55 (1^st^ quintile) (p=0.01). The 5^th^ quintile of CAVI is associated with an increased risk of cardiovascular events [9]. With CAVI, information on the ABI can be acquired simultaneously.

Aim of the study was to analyse cardiovascular risk factors in the group of aged patients with ischemic heart disease. These patients were recruited from previously studied cohort of 171 subjects in whom 7-day-24h-ABPM was performed and which revealed interesting findings [1]. The present study compares the relationships among four different parameters predicting cardiovascular risk: the Night-to-Day ratio, Ambulatory Arterial Stiffness Index (both determined from 7-day ambulatory blood pressure monitoring), Cardio-Ankle Vascular Index, and Ankle-Brachial Index (both determined by vascular screening device VaSera). All parameters were determined twice, at the beginning and at the end of one year period.

## Methods

### Study participants

A group of 20 patients with ischemic heart disease was included in the study. Each of them underwent two 7-day-24h-ABPM sessions (total of 280 24h-ABPM records) which revealed 8,120 BP measurements (29 values per daily record); the first measurement was performed at the onset and the second measurement at the end of this one-year study.

For each of the two 7-day-24h-ABPM records, the ND-R of SBP and DBP and AASI were determined in 24-hour cycle and in 7-day period. CAVI and ABI were always examined at the beginning of each 7-day-24h-ABPM session, at the onset of the study and at its end.

All 20 patients (retired persons – 12 men; 8 women; mean age 67±2.1 yrs; mean BMI 29.3±1.69 kg/m^2^; mean left ventricle ejection fraction 53±3.8 %) suffered from chronic ischemic heart disease and were involved in cardiovascular rehabilitation. All subjects were diagnosed with dyslipidemia (TG above 1.7 mmol/l, LDL above 3.0 mmol/l) and were undergoing the same treatment (beta-blockers, ACE inhibitors, and statins). They were symptomatically stable and their medication was not changed during the whole study. The presence of diabetes was not confirmed in the study subjects. All of them were non-smokers. Their average sleep duration was 7.5 h.

### Measurement methods

The 7-day-24h-ABPM records were obtained with the TM-2430 device from A&D (Tokyo, Japan), based on the cuff oscillometric method. The device automatically records all scheduled measurements around the clock for 7 days, at 30-min intervals between 6:00 am and 10:00 pm and at 60-min intervals between 10:00 pm and 6:00 am. The patients recorded their daily activities, their sleep times, and eventual subjective problems. To make BP measurements objectively comparable among the patients, their sleep/awake times had to be defined. Therefore, the mean nocturnal BP for individual days was calculated between 1:00 and 6:00 am and the mean daytime BP between 10:00 am and 10:00 pm, omitting BP measurements between 7:00 and 9:00 am and between 10:00 pm and 12:00 am. Thus, the sleep and awake time intervals were applied to all patients [1].

CAVI was always measured in the morning in the supine position, using a vascular screening system VaSera VS-1000 device (Fukuda Denshi, Tokyo, Japan). The CAVI measurements were obtained from the brachial and ankle pulse wave forms, SBP and DBP. They were determined using the following formula: CAVI=a {(2ρ/ΔP)×ln (Ps/Pd) PWV2}+b, where a and b are constants applied according to the value derived from the modification of Bramwell Hills, ρ is blood density, ΔP is Ps-Pd, Ps is SBP, Pd is DBP, and PWV is pulse-wave velocity [7]. Electrocardiographic electrodes were attached to the upper arms, and a microphone was placed on the sternal angle for phonocardiography. Cuffs were applied around the upper arms and ankles of the lower legs, bilaterally. After resting for 10 min, the examinations were performed. All measurements were automatically calculated using the VaSera VS-1000.

CAVI value <8 was interpreted as a mild risk of arteriosclerosis, value between 8 and 9 was interpreted as a moderate risk of arteriosclerosis, and value >9 was interpreted as a high risk of arteriosclerosis; CAVI ≥9 was defined as abnormal.

ABI was simultaneously measured using the same equipment and calculated by dividing the ankle SBP by the brachial SBP.

### Statistical analysis

All records of 7-day-24h-ABPM were processed as follows: the mean BP value in day time was obtained as an average from two values measured in particular hour. After omitting BP measurements between 7:00 and 9:00 am and between 10:00 pm and 12:00 am and after including night time measurements, total of 19 mean BP values were obtained per each 24-hours cycle. Then, mean week values and calculated N-DR in every day for both measurements were calculated in the same way. Similarly, AASI was evaluated in days and weeks values in the first and the second examinations.

Checks for normality of the 24-hour ABPM data distribution in individual patients were performed with the Shapiro-Wilk test. When p>0.05, a two-sample paired *t*-test was subsequently used to compare the mean values between two consecutive recordings; in case of p<0.05, the Wilcoxon paired the test was used instead.

Paired *t*-tests were used to compare data between the first and the second examination sessions. The comparison of the ND-R between men and women was performed by the two-sample Student *t*-test assuming inequality of variance.

AASI evaluation was performed using regression analysis (MS Excel). Correlation coefficients (MS Excel) were calculated to determine the strength of the associations.

The frequency of the qualitative distribution according to the SBP and DBP DN-R (D, ND, ED, RD) was evaluated by the independence test in contingency tables followed by Fisher’s test to determine the two-sided probability.

A p-value of less than 0.05 was considered to indicate statistical significance.

## Results

### Night-to-Day Ratio

**Fig. 1:**
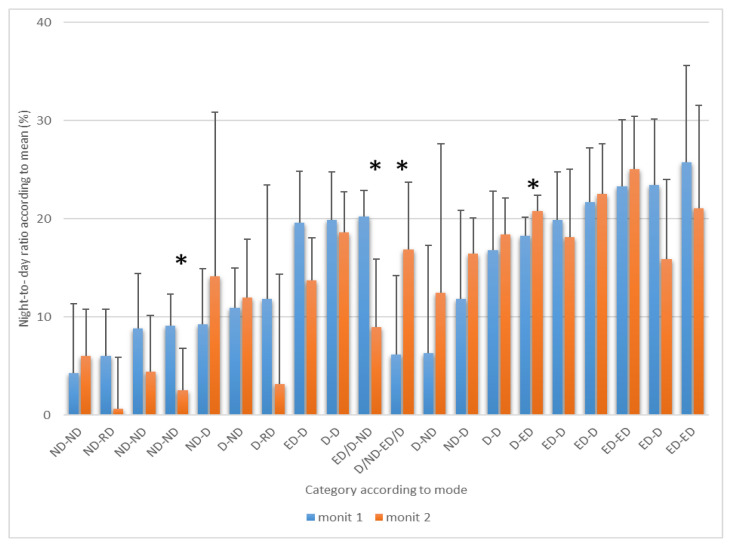
Mean values of Night-to-Day Ratio of SBP during the first and the second 7-day-24h-ABPM in individual patients with chronic ischemic heart disease (n=20). D – dipper 10–20 %, ND – non dipper <10 %, ED – extreme dipper >20 %, RD – reverse dipper <0 %, ED/D; D/ND – the mode cannot be clearly determined, the frequency of ED and D (RD and D; D and ED) was the same, * p<0.05. Only the positive deviation from the average is shown in the graph for greater clarity. Subjects are always shown in the same order (X-axis).

**Fig. 2:**
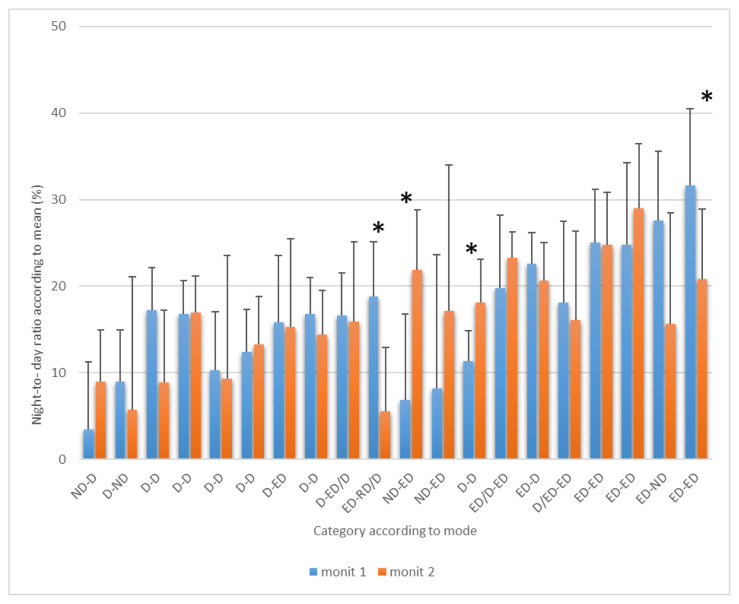
Mean vs. mode of Night-to-Day Ratio of DBP during the first and the second 7-day-24h-ABPM in individual patients with ischemic heart disease (n=20). D – dipper 10–20 %, ND – non dipper <10 %, ED – extreme dipper >20 %, RD – reverse dipper <0 %, ED/D; RD/D; D/ED – the mode cannot be clearly determined, the frequency of ED and D (RD and D; D and ED) was the same, * p<0.05. Only the positive deviation from the average is shown in the graph for greater clarity. Subjects are always shown in the same order (X-axis).

Seven-day/24-hour ABPM allows the determination of the ND-R for each day in every patient. The results of N-DR are summarized in Figure 1 and Figure 2 showing the mean ND-R for SBP and DBP across the 7 days for each patient (error bars represent the standard deviation, SD). The mean value of N-DR for SBP (± SEM) is 14.4±1.55 % in the fist examination and 13.4±1.6 % in the second examination, and 15.8±1.7 % in the first examination and 16.5±1.3 % in the second examination for DBP in all subjects (Table 1). The most interesting finding is that the individual variability of ND-R is great.

The patient ID numbers on the X-axis are substituted by the mode according to the dipping category for the first and the second monitoring sessions (separated by a dash). The ND-R is shown on the Y-axis, with tick marks every 10 %, so the mean value represented by the column defines the ND-R as a mean over 7 days. For example, the dipper category is displayed by all columns in the interval 10 % to 20 %. This result may not be identical to the parameter presented on the X-axis where the mode value is given.

Figure 1 shows the mean ND-R results of the 7-day-24h ABPM for SBP during the first and second monitoring sessions for each individual (n=20), compared to the mode of the dipping category. From the 40 average ND-R values (columns), 18 were classified as dippers, 13 as non-dippers, and 9 as extreme dippers. However, due to the high variability (represented by the SD), the results could be also interpreted as 13 dippers, 2 non-dippers, and 25 extreme dippers. It is more objective to classify the patients into the corresponding categories according to the model (6), the value of which is informatively shown below the X-axis: 14 dippers (35 %), 12 non-dippers (30 %), 9 extreme dippers (22.5 %), and 2 reverse dippers (5%); 3 (7.5 %) subjects couldn’t be clearly classified. A risk assessment was found in 65 % of patients. Statistically significant differences between the first and the second monitoring sessions were demonstrated due to high variability only in 4 patients (4 p<0.019, 10 p<0.016, 11 p<0.003, and 15 p<0.037).

Figure 2 shows the mean ND-R results of the 7-day-24h ABPM for DBP during the first and the second monitoring sessions for each individual (n=20), compared to the mode of the dipping category. From the 40 average ND-R values (columns), 21 were classified as dippers, 9 as non-dippers, and 11 as extreme dippers. However, due to the high variability (represented by the SD), the results could be also interpreted as 11 dippers, 0 non-dipper, and 29 extreme dippers. The classification into the corresponding categories according to the model (6) revealed that in our studied group 17 dippers (42.5 %), 5 non-dippers (12.5 %), 14 extreme dippers (35 %), and 0 reverse dippers were found; 4 (10 %) individuals couldn’t be clearly classified. A risk assessment was found in 57.5 % of patients. Statistically significant differences between the first and second monitoring sessions were demonstrated due to high variability in only 4 patients (10 p<0.004, 11 p<0.006, 13 p<0.014, and 20 p<0.047).

A statistically significant difference between the first and the second monitoring sessions in the 7-day mean ND-R of both SBP and DBP was found only in one patient (No. 11). A comparison between the first and the second monitoring sessions is shown in Table 1. The mean ND-R difference for SBP between the first and the second monitoring sessions was −1.05 % (SEM 1.257 %); it was −0.49 % (SEM 1.65 %) in men and −2.09 % (SEM 1.98 %) in women. However, the difference between men and women were not statistically significant (p=0.546). There is no association (r=−0.050) between the time elapsed between the first and the second monitoring sessions and the difference in nocturnal SBP drop.

The mean ND-R difference for DBP between the first and the second monitoring sessions was 0.67 % (SEM 1.566 %); it was −0.85 % (SEM 1.81 %) in men and 3.49 % (SEM 2.834 %) in women. Again, as in case of SBP, the differences between men and women were not statistically significant (p=0.223). There is no association (r=−0.005) between the time elapsed between the first and the second monitoring sessions and the difference in nocturnal DBP drop.

The decrease in mean ND-R for SBP from 14.4 % to 13.4 % is not significant (p=0.425). The increase in mean ND-R for DBP from 15.8 % to 16.5 % is not significant (p=0.643).

### Ambulatory Arterial Stiffness Index

Mean value (±SEM) of AASI evaluated from 7-days-24h ABPM in the first examination in the whole group was 0.460±0.031 and 0.462±0.021 in the second examination (Table 1).

**Fig. 3:**
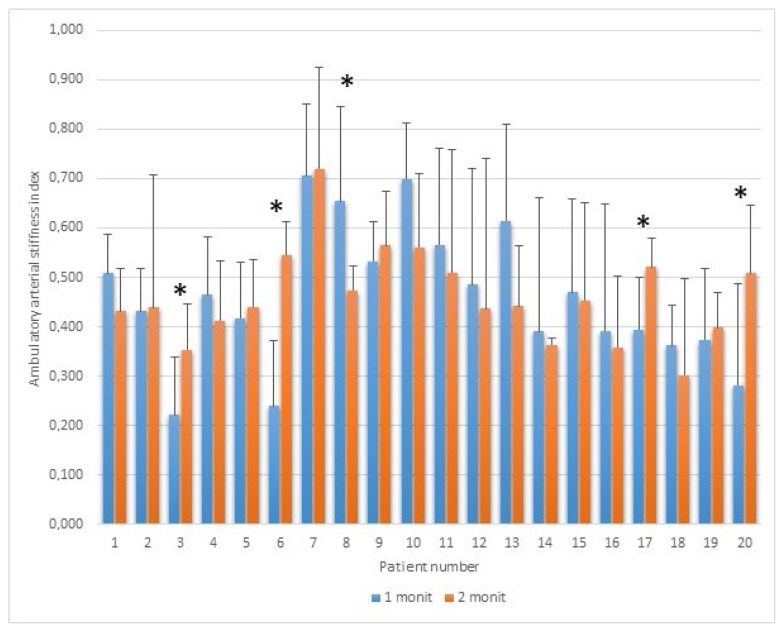
Ambulatory arterial stiffness index (AASI) during the first and the second 7-day-24h-ABPM in individual patients with chronic ischemic heart disease (n=20). * p<0.05. Only the positive deviation from the average is shown in the graph for greater clarity. Subjects are always shown in the same order (X-axis).

Figure 3 shows the mean AASI results of the 7-day-24h ABPM during the first and the second monitoring sessions for each individual (n=20). The 7-day AASI mean exceeding the risk limit of 0.7 was found only in one patient (No. 7) during both sessions. However, due to the high variability represented by the SD, over-limit results (>0.7) could include 11 days of monitoring. The stricter limit of 0.5 (used for people younger than subjects included in this study) was exceeded in 12 cases. Statistically significant differences between the first and the second monitoring sessions were found in only 5 patients (3 p<0.042, 6 p<0.0004, 8 p<0.046, 17 p<0.021, and 20 p<0.034) due to the high variability in daily estimates.

The small increase in AASI between the two sessions was not significant (p=0.959, Table 1). Both mean values (corresponding to the first and the second monitoring sessions) are lower than 0.5. However, the maxima exceed 0.7 (Fig. 3).

### Cardio-Ankle Vascular Index and Ankle-Brachial Index

CAVI and ABI were determined once during each monitoring session (n=20 each) after a period of one year. The mean (± SEM) value in the whole group was 7.8±0.3 in the first examination and 7.6±0.2 in the second examination, respectively; however, this decrease was not significant (p=0.336, Table 1). Mean values during both monitoring sessions are lower than 9.0, maxima reach 11.6.

According to usual classification, overall (irrespective of the monitoring session) CAVI was <8.0 in 76.3 % of patients, between 8 and 9 in 8.7 % of patients, and >9.0 in 15 % of patients. Thus, CAVI results suggest that 23.7 % of patients are at increased cardiovascular risk.

A recent risk classification divides CAVI into 5 quintiles (16). In accordance with this classification, 63.8 % of patients were included in the first quintile (CAVI<7.55), 13.7 % of patients in the second quintile (CAVI 7.6–8.2), 5 % of patients in the third quintile (CAVI 8.25–8.8), 2.5 % of patients in the fourth quintile (CAVI 8.85–9.45), and 15 % of patients in the fifth quintile (CAVI>9.5).

### Ankle-Brachial Index

Mean values (± SEM) of ABI in the whole group was 1.13±0.01 in the first examination and 1.11±0.02 in the second examination. The decrease in ABI from 1.13 to 1.11 between the two monitoring sessions was not significant (p=0.462, Table 1). The minima were not lower than 0.9. Overall (irrespective of monitoring session) ABI was between 1.01 and 1.4 (considered normal) in 92.5 % of patients and between 0.9 and 1.0, which represents an acceptable range (considered borderline), in 7.5 % of patients.

### Correlation between results from different methods

Table 1 summarizes the results of all methods used to assess the cardiovascular risk (ND-R, AASI, CAVI, and ABI). The mean ND-R of SBP and DBP and AASI were calculated from separate daily spans from the 7-day-24h ABPM records for all patients (n=140 for each monitoring session).

Table 2 shows the correlation coefficients comparing results from all methods used in this study (n=200, 7-day-24h-ABPM records obtained in 20 patients who were monitored for 7 days, twice, about 11 months apart).

Moderate reliability correlation was observed between ND-R for SBP and DBP (r=0.752). In four patients, the correlations were extremely low. The test of independence in the contingency table (χ^2^=10.248) indicates that the qualitative classification of 20 patients according to the ND-R calculated for each of the 14 observed days in the categories according to the mode (D, ND, ED, R) depends on the variable (SBP or DBP) (p=0.042).

The correlation coefficients between AASI and ND-R for SBP or DBP were very low (r=−0.153 and r=−0.318, respectively). Correlations evaluated for individual patients exceeded the value r=−0.8 (good reliability) only in four out of 40 cases, always for only one monitoring session: patient No. 7 (the first monitoring session, SBP; r=−0.818), patient No. 9 (the second monitoring session, DBP; r=−0.827), patient No. 13 (the first monitoring session, DBP; r=−0.826), and patient No. 17 (the first monitoring session, DBP; r=−0.890).

## Discussion

The high variability of BP – both intraindividual and interindividual – is generally accepted fact [10]. However, highly varying data represent a challenge during statistical analysis. Reduction of the disturbing impact of high variability of biological data can be ensured either by the highest possible homogeneity of the individuals in a small monitored group or by a large range of inhomogeneous groups (e.g., a high number of measurements or a high number of monitored individuals).

Both abovementioned approaches were employed in the present study. First, highly homogeneous group of 20 patients was studied. There were no differences between the sexes found. Correlations between the two monitoring sessions were not statistically significant, and differences in nocturnal BP drops did not depend on the time elapsed between consecutive ABPM records (r=−0.050 for SBP and r=−0.005 for DBP, respectively). The mean and mode values reported in Table 1 are close, indicating a normal distribution.

However, the high variability of the parameters we monitored would require optimal monitoring of thousands of patients (e.g. for AASI 58533 individuals – Required Sample Size (N) at a power setting of 0.8). The number of individuals in our study is therefore not statistically sufficient and the informative power of the conclusions is limited. The low number of individuals in the study group does not allow examining specific parameters such as the presence or absence of diabetes, control status, plasma LCL-C concentration and smoking history. These parameters were within physiological values in the patients in our group, they were non-smokers.

In contrast, a considerable number of BP measurements (8120 records) from repeated 7-day-24h-ABPM was evaluated, which represents an important methodological tool for the objectification of BP assessment. Since 7-day-24h-ABPM always covers the entire week, the possible influence of working days and weekends is suppressed. Furthermore, it partially eliminates the novelty effect, the white coat syndrome, and the influence of activities that cause extreme BP fluctuations.

At present, hypertension management guidelines do not reflect BP variability. The possible reason is the lack of available evidence on its clinical relevance. So far available information is based on heterogeneous studies with limited standardization of methods for BP variability assessment [11].

Otsuka *et al.* advocate the use of 7-day-24h records of BP as an effective tool for finding masked hypertension, masked morning surge, and other rhythm abnormalities. Chronobiological aspects must also be included in the assessment [12,13].

In the present study, ND-R for SBP correlated weakly (moderate reliability) with ND-R for DBP (r=0.752; Table 2). This was the reason for establishing the ND-R for DTK as well. Dipping categorization (D, ND, ED, and R) according to the ND-R mode depends on whether it is based on SBP or DBP. The result of the independence test in the contingency tables also agrees with this fact (p=0.042).

The high agreement in the outcomes from ND-R classification between the 7-day-24h-ABPM and from individual 24-hour days records ranges between 59 % and 62 % in each studied patient. Only in a few cases it does reach an agreement of 0 % or 100 % [1].

The Ambulatory Arterial Stiffness Index (AASI) is a novel approach how to evaluate arterial stiffness; it independently predicts cardiovascular mortality, even in normotensive subjects [2]. When hypertensive patients are divided into four dipping classes with respect to the extent of nocturnal BP reduction (extreme dipper, dipper, non-dipper, and reverse dipper), the AASI may be able to estimate different degrees of arterial stiffness resulting in a different extent of organ damage [3]. The AASI is not originally calculated and validated from 7 days of BP monitoring, but only from 24 h of BP monitoring [2].

In order to mitigate various influences on the high variability of BP, we determined ND-R and AASI from 7-day-24h-ABPM monitoring, repeated after one year.

AASI appears to be independently associated with age, SBP and pulse pressure, and inversely with the nocturnal drop in SBP and DBP [14]. Based on the results from the present study, the hypothesis that AASI is unable to estimate the arterial stiffness in older hypertensive subjects with a high burden of organ and vascular damage and several comorbidities [15] is confirmed, probably because the nocturnal reduction of BP is the main determinant of AASI, being more powerful than arterial stiffness itself [3,16].

There is controversy to what extent AASI derived from ABPM recordings reflects arterial stiffness or is affected by other parameters (arterial distensibility, peripheral resistance, heart rate, maximal cardiac elastance, and venous filling pressure) [17]. Therefore, various arterial stiffness indexes have been proposed and used in research settings [18]. CAVI has been introduced as a BP-independent measure of arterial stiffness as CAVI has been derived from the concept of β-stiffness index [19]. Several studies have verified the BP independence of CAVI [20,21]. CAVI is significantly associated with cfPWV (r=0.74) and baPWV (r=0.82).

In order to estimate cardiovascular risk, CAVI can be evaluated in two ways. According to the commonly used classification [20], CAVI less than 8.0 is considered normal, whereas a value less than 9.0 (but more than or equal to 8.0) is considered borderline, and a value of 9.0 or more leads to the diagnosis of suspected arteriosclerosis or cardiovascular risk. A more recent classification divides risks into five quintiles (quintile 1, ≤7.55; quintile 2, 7.60–8.20; quintile 3, 8.25–8.80; quintile 4, 8.85–9.45; and quintile 5, ≥9.50). The cumulative incidence of adverse cardiovascular events is significantly higher for CAVI>9.5 (quintile 5) than for CAVI<7.55 (quintile 1) (p=0.01). The quintile 5 of CAVI is associated with an increased risk of cardiovascular events [9]. With CAVI, information on the ABI can be acquired simultaneously.

Numerous studies have demonstrated the relationship of CAVI with atherosclerosis and obstructive artery disease. CAVI is independently associated with the progression and severity of coronary atherosclerosis [22,23].

CAVI reflects both the organic and functional stiffness of the arterial wall. It is not a pure indicator of organic changes in the arterial wall due to atherosclerosis or aging [24]. CAVI is affected by the condition of smooth muscle in the arteries, which manifests itself in changes in arterial tone [9]. An increase in sympathetic tone is a sign of, for example, resistant hypertension or heart failure [25], when CAVI determination is advantageous.

CAVI cannot be evaluated in case of low values of the ABI index (<0.9). In such patients, the atherosclerotic changes in the peripheral arteries are so extensive that the determination of CAVI is not valid [7]. In such cases, the conventional determination of pulse wave velocity (PWV) [26] can be recommended.

ABI index values less than 0.9 are diagnostic for peripheral artery disease, despite the fact that more than 80 % of individuals with this finding have no clinical manifestations [27]. Moreover, the presence of decreased ABI is associated with a higher incidence of coronary and cerebrovascular complications and a higher risk of death due to the increase in cardiovascular mortality [28].

PWV well correlates with the presence and extent of coronary, cerebral and carotid atherosclerosis. However, interpreting of PWV results must be done with caution since numerous clinical factors affect PWV, markedly BP [29].

Findings from the different methods used in this study did not correlate mutually (Table 2). The highest correlation coefficient of −0.318 was found between AASI and ND-R for DBP. The ND-R, AASI, CAVI, and ABI cannot replace each other.

Since many patients facing fatal CVD events such as sudden cardiac death, myocardial infarction, or stroke do not have prior symptoms or warning signs [30,31], it is of high importance to detect subclinical BP changes and atherosclerosis at their early stages, and thus to identify individuals who are at high risk for future cardiovascular diseases.

## Conclusions

Taking in consideration high incidence and mortality due to cardiovascular diseases, it is of high importance to search for potential improvement of diagnosing these disorders. Common disease, especially (but not only) among aged patients, is atherosclerosis. However, it is not always diagnosed properly. This study showed the high individual variation of cardiovascular risk parameters. Repeating the 7-day-24h-ABPM approximately 1 year apart (unless there is a change in medication or in clinical symptoms) revealed a significantly different results of the ND-R and AASI, which can be expected in approximately 25 % of patients. It was also shown that ND-R, AASI, CAVI, and ABI did not correlate mutually. Among the used approaches, determination of the ND-R for SBP showed the highest methodological sensitivity. In patients included in this study, 65 % were assessed as having an increased risk. Taking together, when evaluating eventual cardiovascular risk of a patient, complex approach is needed and use of several methods is beneficial.

## Figures and Tables

**Table 1:** Results of five cardiovascular risk methods used to assess arterial stiffness in 20 patients: comparison between the first and the second monitoring sessions

	ND-R SBP		ND-R DBP		AASI		CAVI		ABI	
*Monitoring periods*	1	2	1	2	1	2	1	2	1	2
*MEAN*	14.4	13.4	15.8	16.5	0.460	0.462	7.8	7.6	1.13	1.11
*SEM*	1.55	1.60	1.71	1.33	0.0312	0.0211	0.30	0.27	0.017	0.020
*MEDIAN*	14.1	14.6	16.6	16.2	0.448	0.441	7.4	7.4	1.12	1.11
*MIN*	4.0	0.7	3.3	4.8	0.222	0.303	5.9	5.5	0.96	0.95
*MAX*	25.4	24.9	30.7	28.8	0.706	0.720	11.6	10.4	1.26	1.30

ND-R – Night-to-Day Ratio (%), AASI – Ambulatory Arterial Stiffness Index, CAVI – Cardio-Ankle Vascular Index, ABI – Ankle-Brachial Index.

**Table 2:** Interdependence of the results from different methods to assess arterial stiffness irrespective of monitoring session (n=280 daily ABPM records).

r	ND-R SBP	ND-R DBP	AASI	CAVI	ABI
*ND-R SBP*	1				
*ND-R DBP*	0.752	1			
*AASI*	−0.153	−0.318	1		
*CAVI*	−0.099	−0.029	0.165	1	
*ABI*	0.208	0.260	−0.033	−0.126	1

ND-R – Night-to-Day Ratio, AASI – Ambulatory Arterial Stiffness Index, CAVI – Cardio-Ankle Vascular Index, ABI – Ankle-Brachial Index, r – Correlation coefficient.
